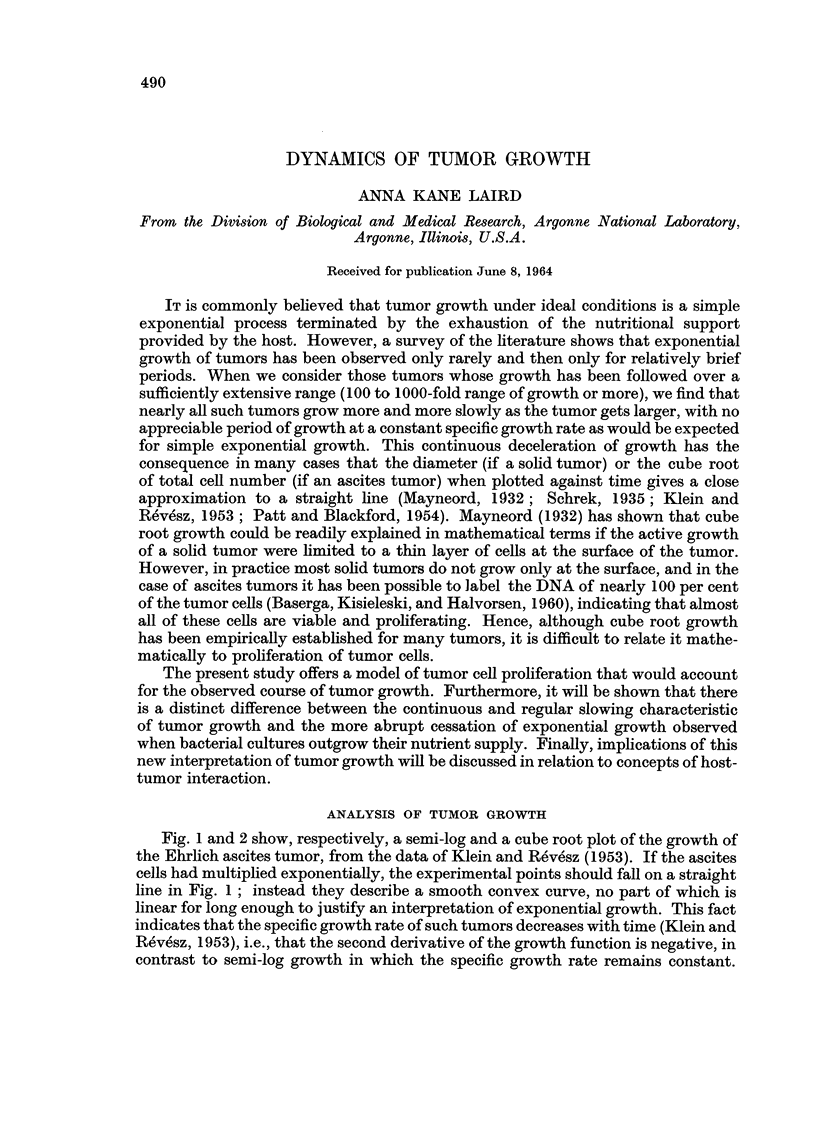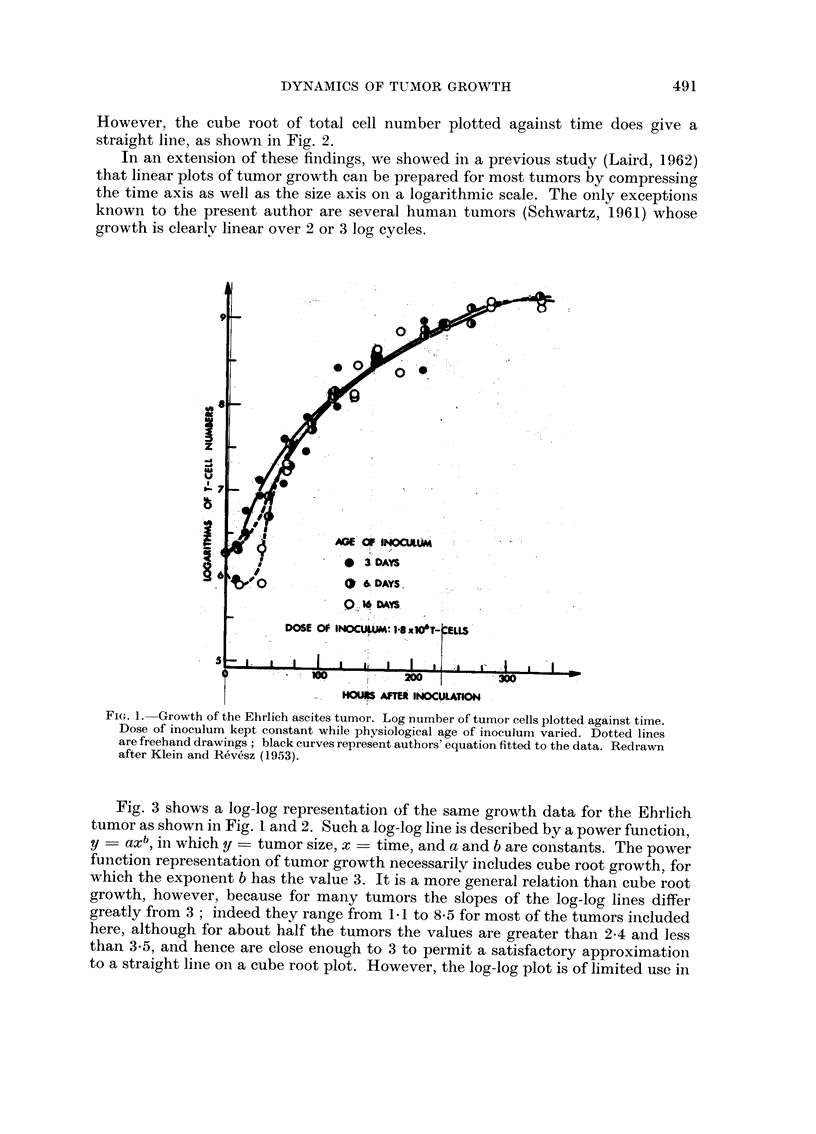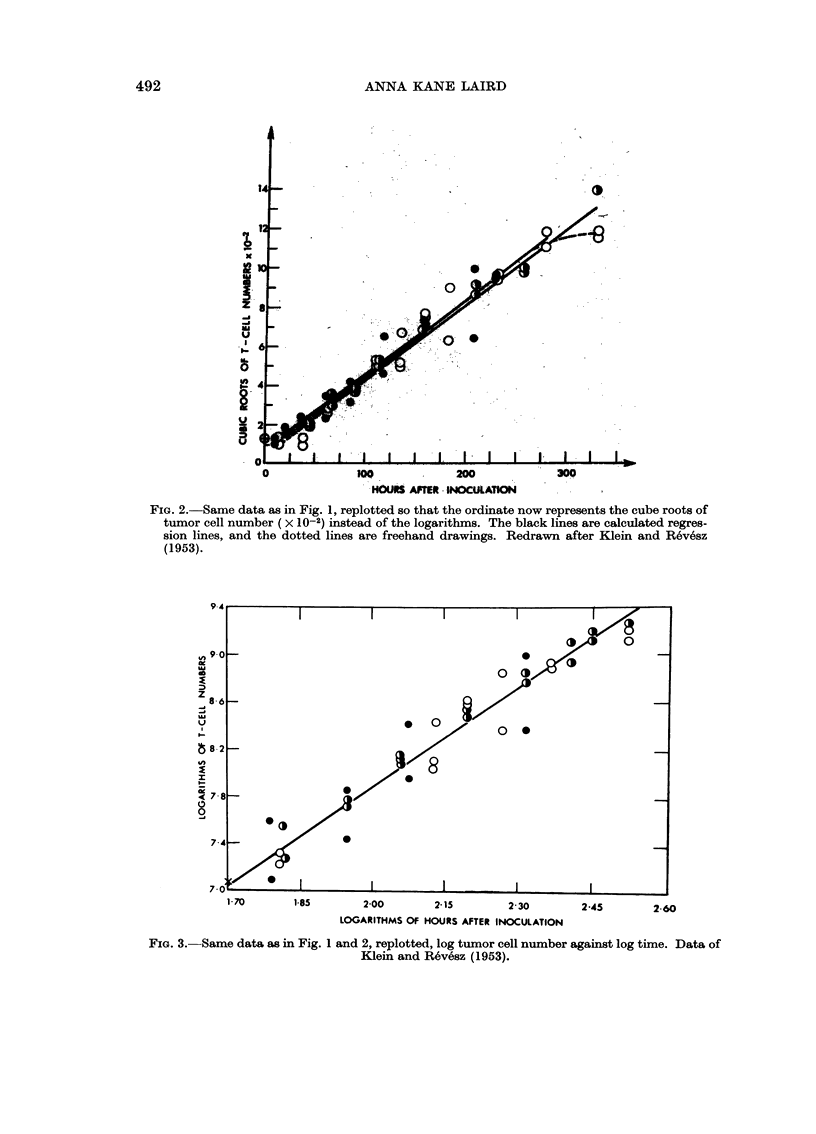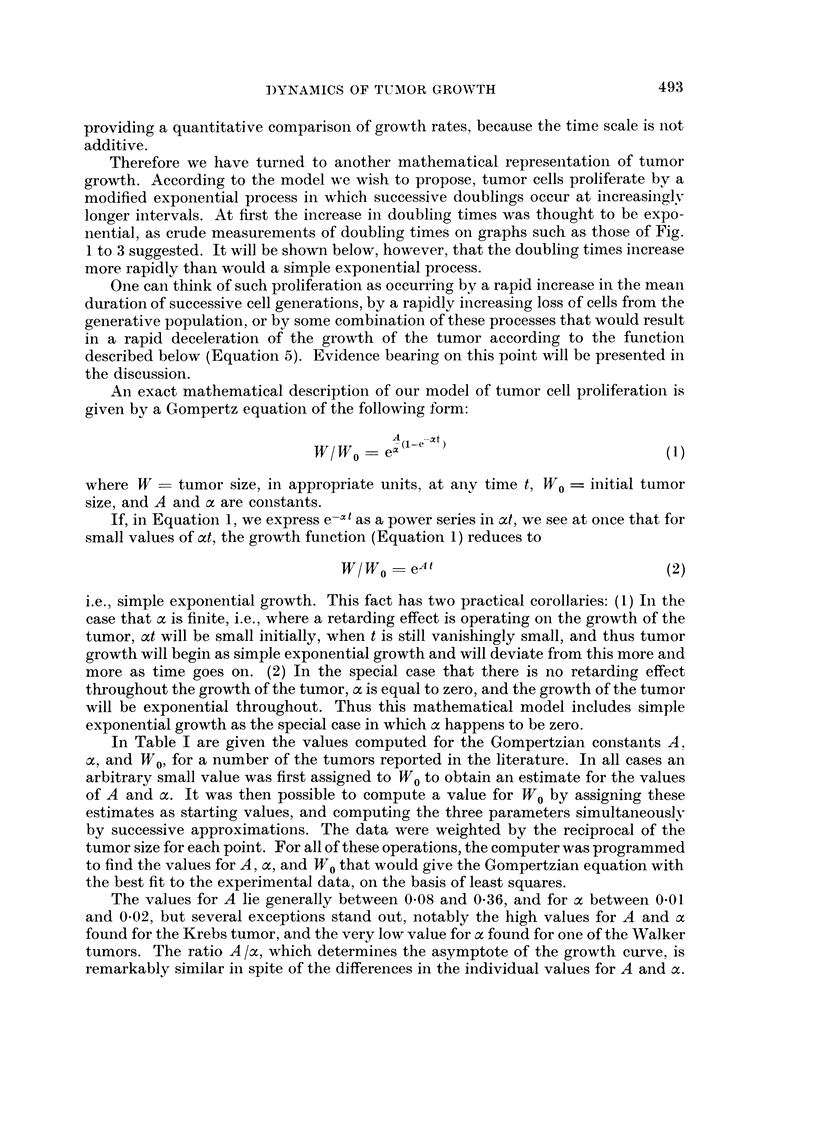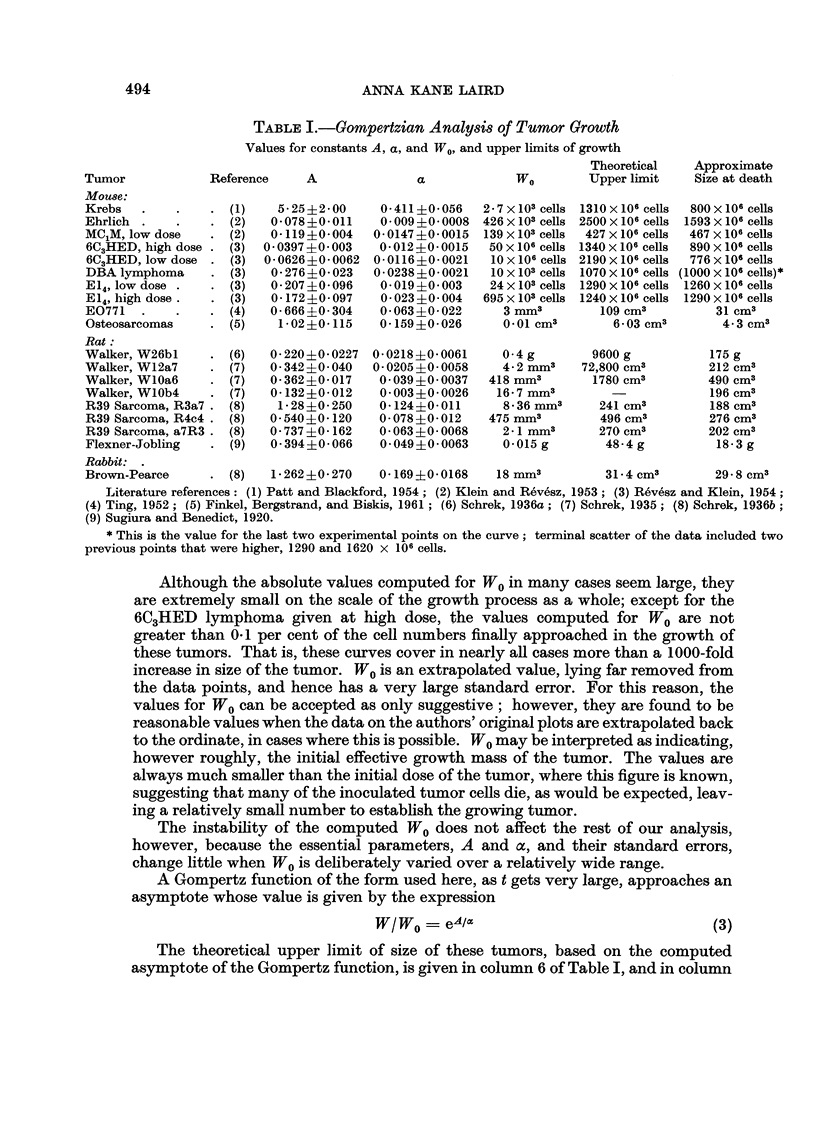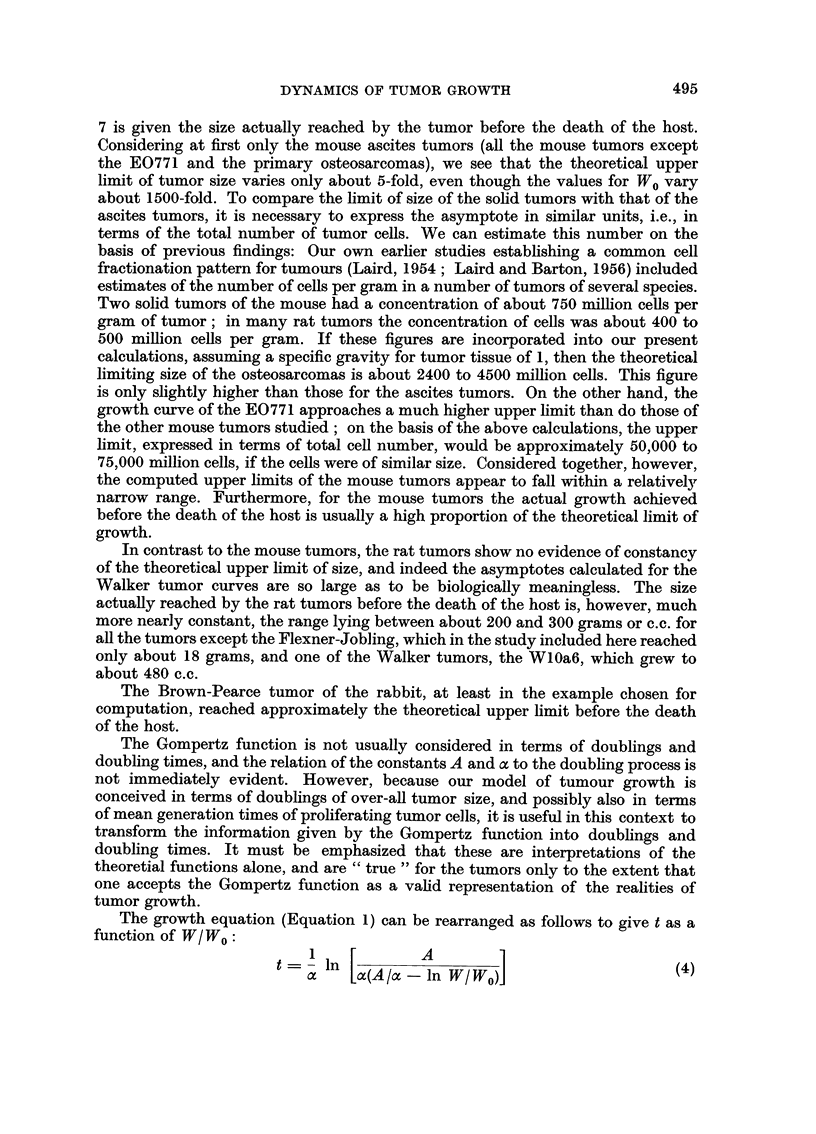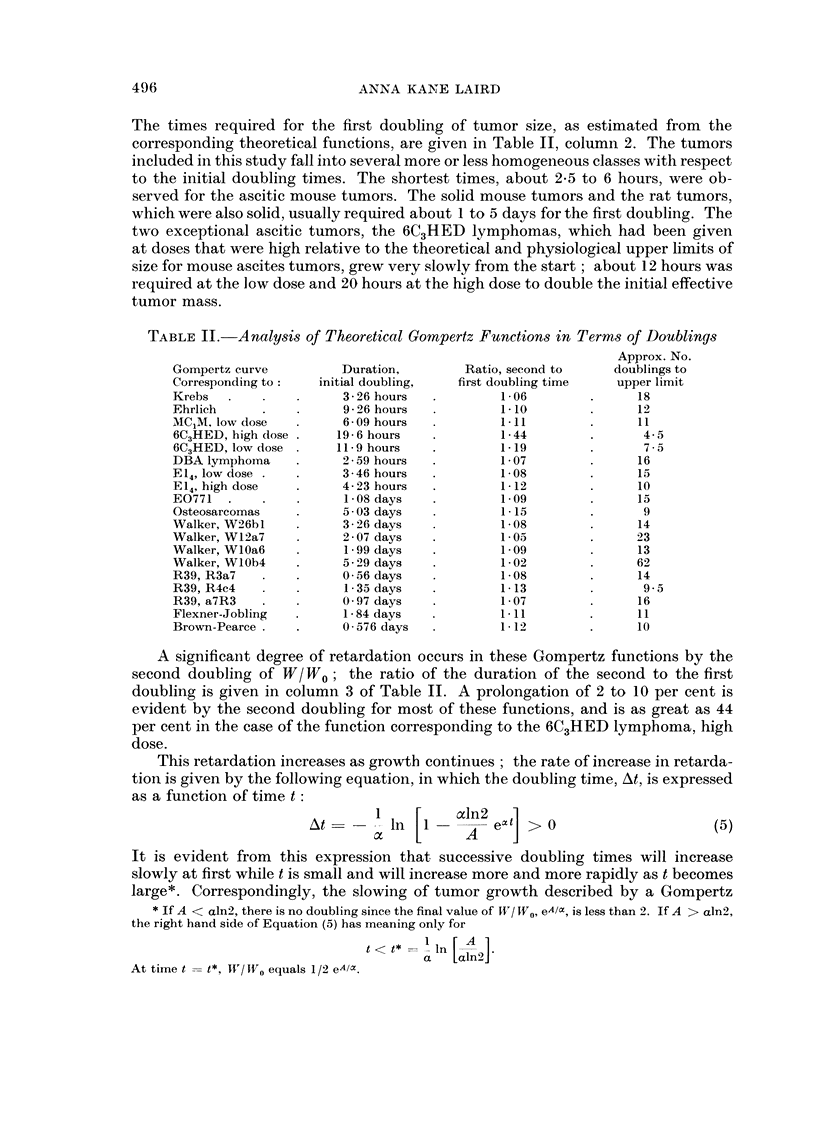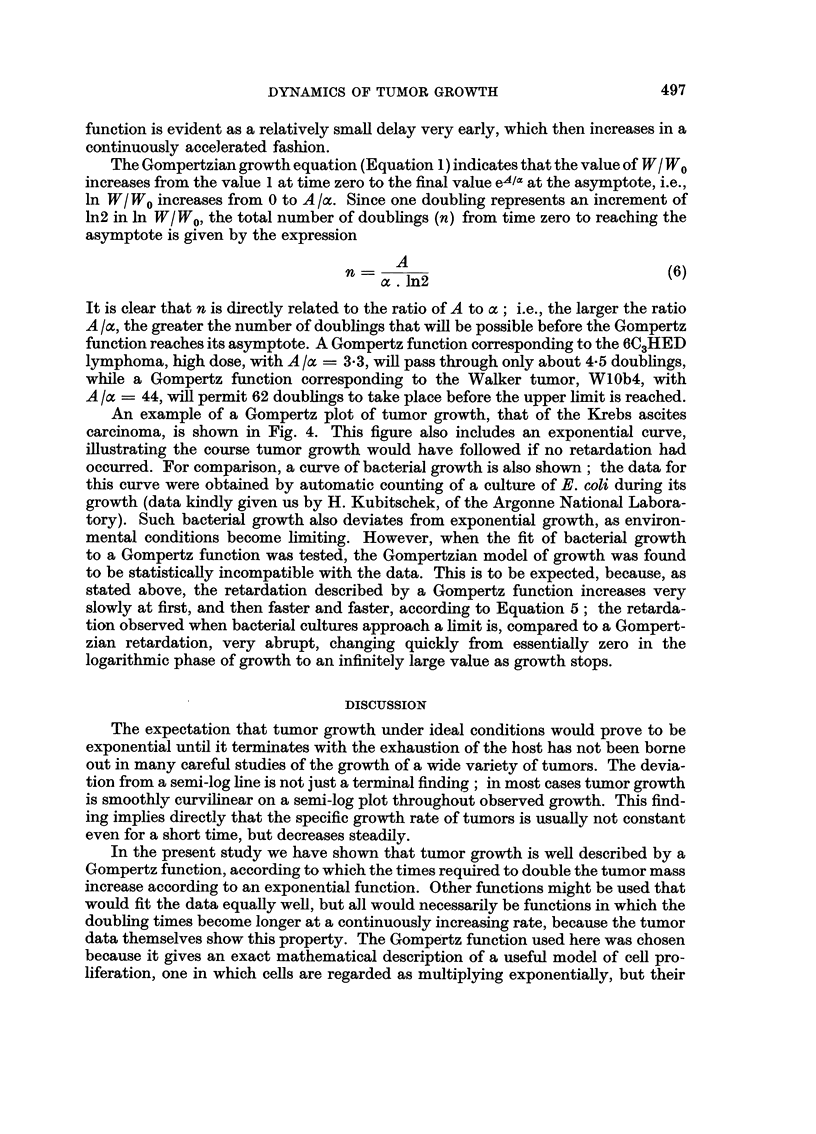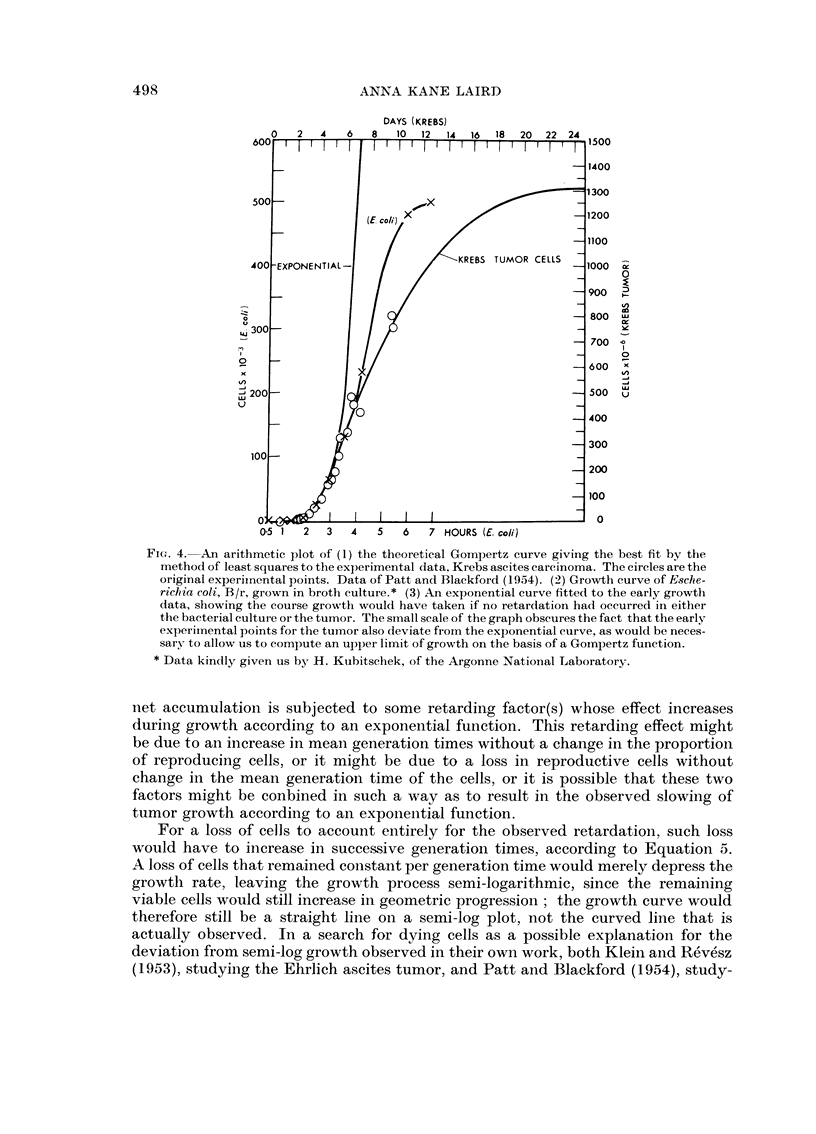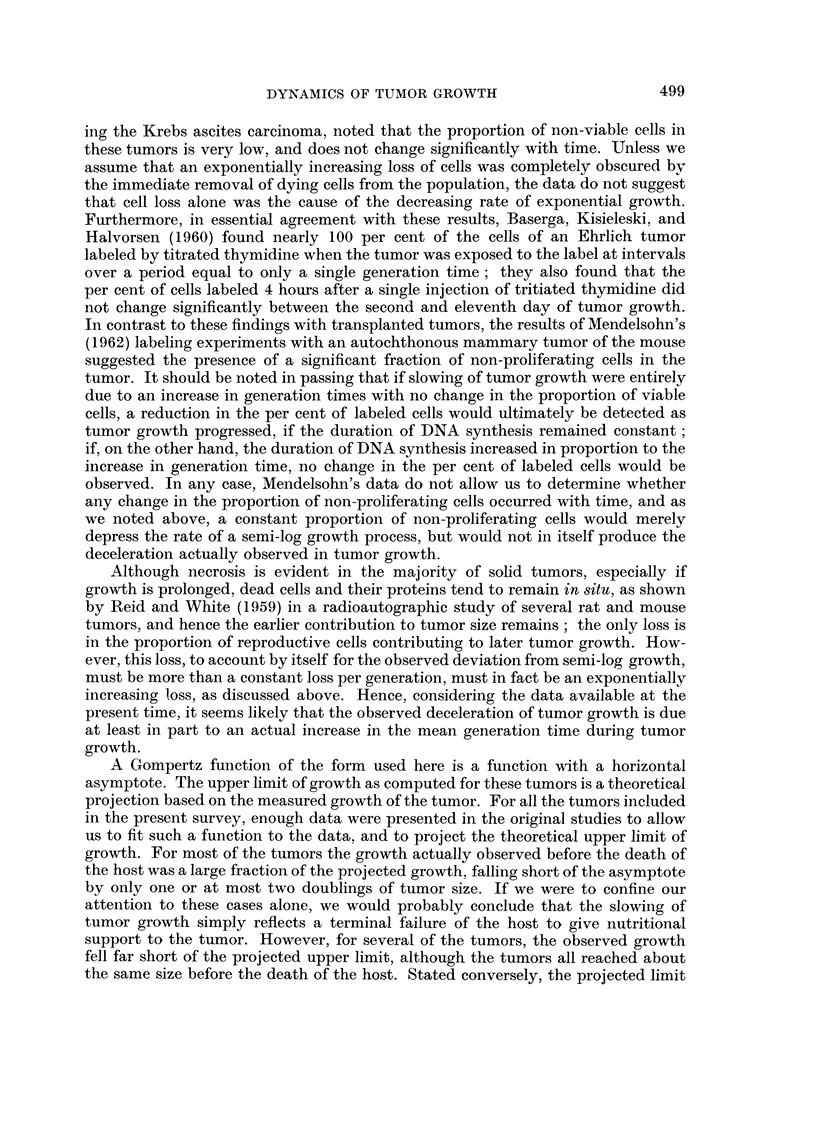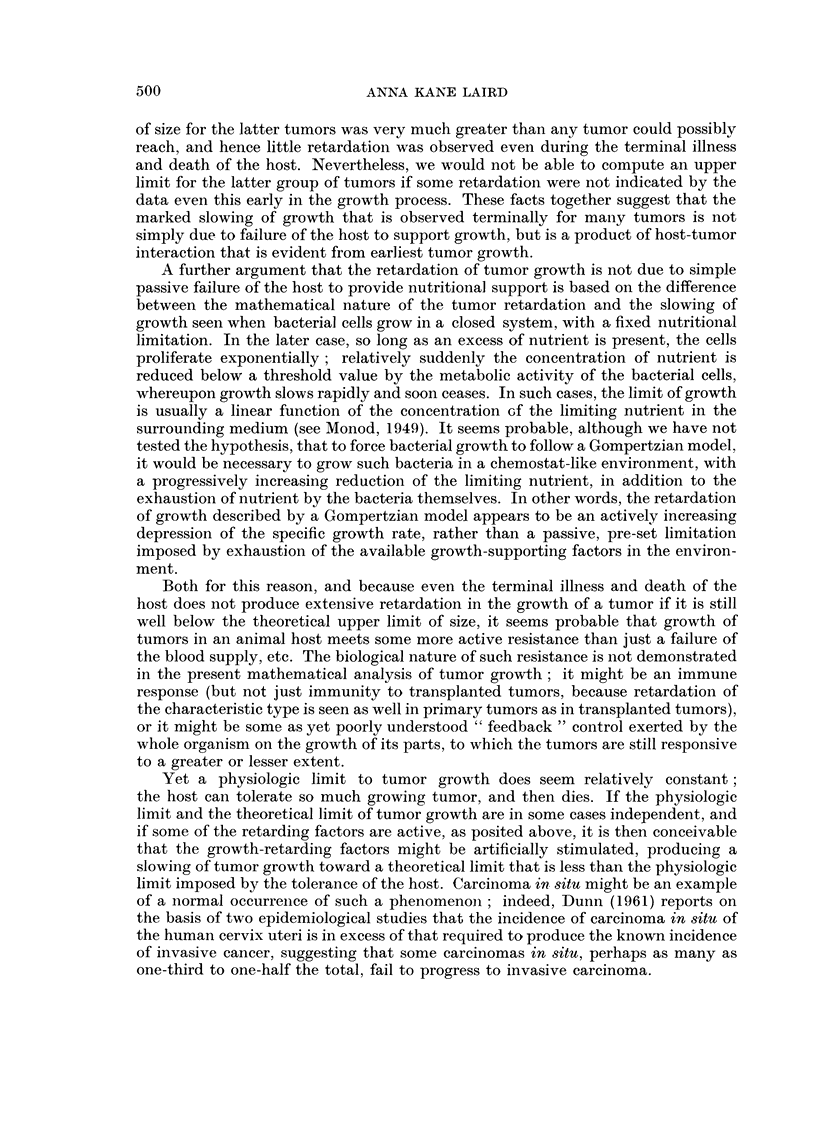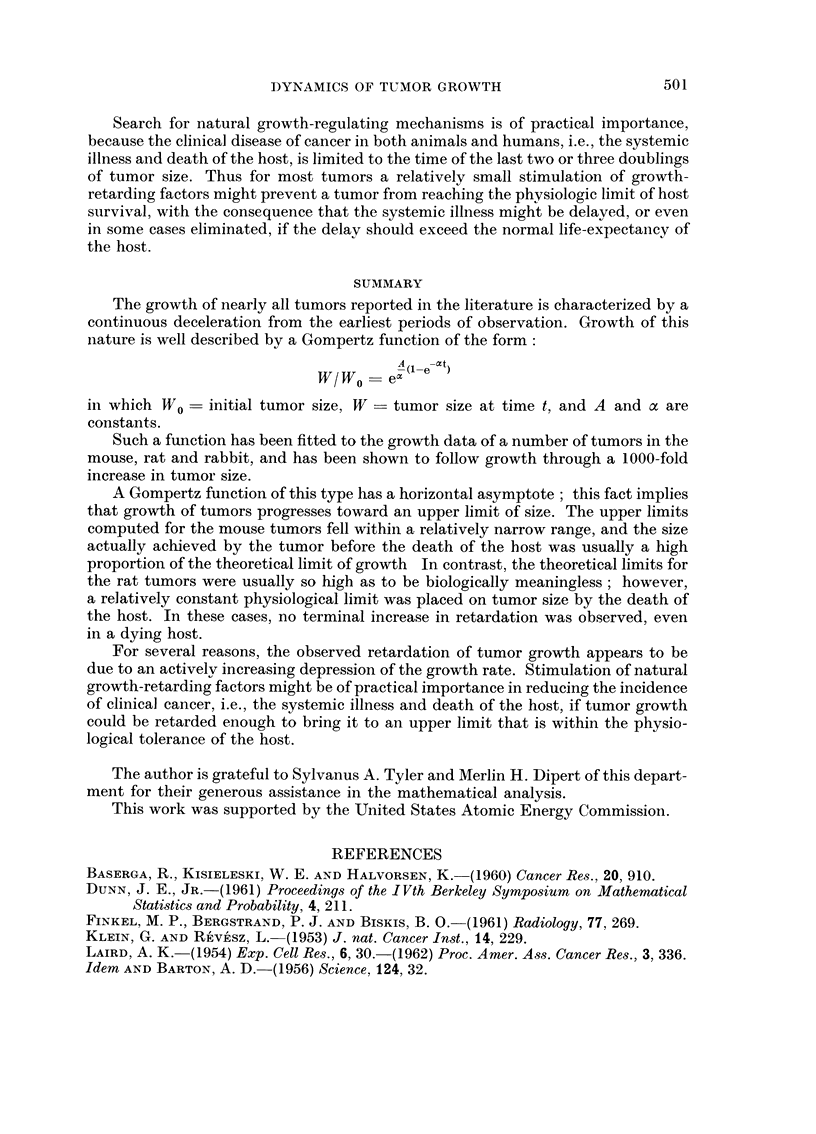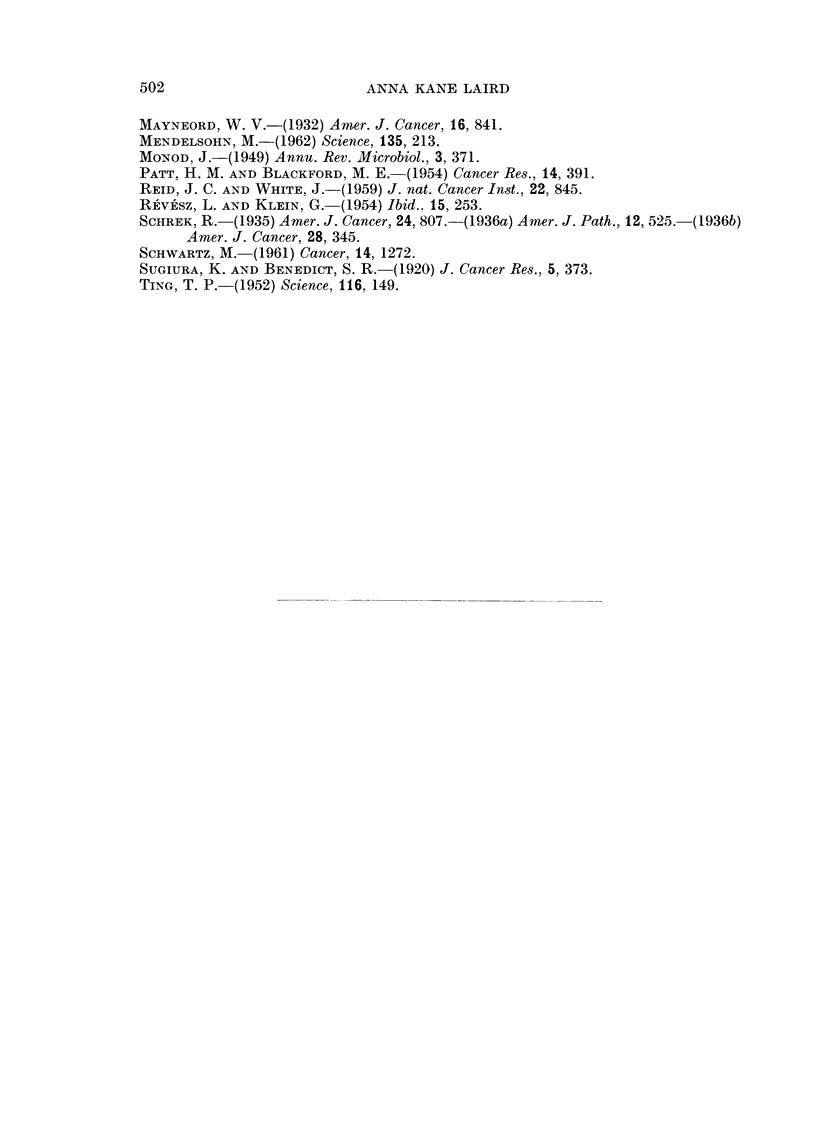# Dynamics of Tumour Growth

**DOI:** 10.1038/bjc.1964.55

**Published:** 1964-09

**Authors:** Anna Kane Laird


### DYNAMICS OF TUMOR GROWTH

ANNA KANE LAIRD

*From the Division of Biological and Medical Research, Argonne National Laboratory, Argonne, Illinois, U.S.A.*

Received for publication June 8, 1964

It is commonly believed that tumor growth under ideal conditions is a simple exponential process terminated by the exhaustion of the nutritional support provided by the host. However, a survey of the literature shows that exponential growth of tumors has been observed only rarely and then only for relatively brief periods. When we consider those tumors whose growth has been followed over a sufficiently extensive range (100 to 1000-fold range of growth or more), we find that nearly all such tumors grow more and more slowly as the tumor gets larger, with no appreciable period of growth at a constant specific growth rate as would be expected for simple exponential growth. This continuous deceleration of growth has the consequence in many cases that the diameter (if a solid tumor) or the cube root of total cell number (if an ascites tumor) when plotted against time gives a close approximation to a straight line (Mayneord, 1932 ; Schrek, 1935 ; Klein and Révész, 1953 ; Patt and Blackford, 1954). Mayneord (1932) has shown that cube root growth could be readily explained in mathematical terms if the active growth of a solid tumor were limited to a thin layer of cells at the surface of the tumor. However, in practice most solid tumors do not grow only at the surface, and in the case of ascites tumors it has been possible to label the DNA of nearly 100 per cent of the tumor cells (Baserga, Kisieleski, and Halvorsen, 1960), indicating that almost all of these cells are viable and proliferating. Hence, although cube root growth has been empirically established for many tumors, it is difficult to relate it mathematically to proliferation of tumor cells.

The present study offers a model of tumor cell proliferation that would account for the observed course of tumor growth. Furthermore, it will be shown that there is a distinct difference between the continuous and regular slowing characteristic of tumor growth and the more abrupt cessation of exponential growth observed when bacterial cultures outgrow their nutrient supply. Finally, implications of this new interpretation of tumor growth will be discussed in relation to concepts of host-tumor interaction.

### ANALYSIS OF TUMOR GROWTH

Fig. 1 and 2 show, respectively, a semi-log and a cube root plot of the growth of the Ehrlich ascites tumor, from the data of Klein and Révész (1953). If the ascites cells had multiplied exponentially, the experimental points should fall on a straight line in Fig. 1 ; instead they describe a smooth convex curve, no part of which is linear for long enough to justify an interpretation of exponential growth. This fact indicates that the specific growth rate of such tumors decreases with time (Klein and Révész, 1953), i.e., that the second derivative of the growth function is negative, in contrast to semi-log growth in which the specific growth rate remains constant.

However, the cube root of total cell number plotted against time does give a straight line, as shown in Fig. 2.

In an extension of these findings, we showed in a previous study (Laird, 1962) that linear plots of tumor growth can be prepared for most tumors by compressing the time axis as well as the size axis on a logarithmic scale. The only exceptions known to the present author are several human tumors (Schwartz, 1961) whose growth is clearly linear over 2 or 3 log cycles.

FIG. 1.—Growth of the Ehrlich ascites tumor. Log number of tumor cells plotted against time. Dose of inoculum kept constant while physiological age of inoculum varied. Dotted lines are freehand drawings ; black curves represent authors' equation fitted to the data. Redrawn after Klein and Révész (1953).

Fig. 3 shows a log-log representation of the same growth data for the Ehrlich tumor as shown in Fig. 1 and 2. Such a log-log line is described by a power function, $y = ax^b$, in which $y$ = tumor size, $x$ = time, and $a$ and $b$ are constants. The power function representation of tumor growth necessarily includes cube root growth, for which the exponent $b$ has the value 3. It is a more general relation than cube root growth, however, because for many tumors the slopes of the log-log lines differ greatly from 3 ; indeed they range from 1·1 to 8·5 for most of the tumors included here, although for about half the tumors the values are greater than 2·4 and less than 3·5, and hence are close enough to 3 to permit a satisfactory approximation to a straight line on a cube root plot. However, the log-log plot is of limited use in

FIG. 2.—Same data as in Fig. 1, replotted so that the ordinate now represents the cube roots of tumor cell number ( $\times 10^{-2}$) instead of the logarithms. The black lines are calculated regression lines, and the dotted lines are freehand drawings. Redrawn after Klein and Révész (1953).

FIG. 3.—Same data as in Fig. 1 and 2, replotted, log tumor cell number against log time. Data of Klein and Révész (1953).

providing a quantitative comparison of growth rates, because the time scale is not additive.

Therefore we have turned to another mathematical representation of tumor growth. According to the model we wish to propose, tumor cells proliferate by a modified exponential process in which successive doublings occur at increasingly longer intervals. At first the increase in doubling times was thought to be exponential, as crude measurements of doubling times on graphs such as those of Fig. 1 to 3 suggested. It will be shown below, however, that the doubling times increase more rapidly than would a simple exponential process.

One can think of such proliferation as occurring by a rapid increase in the mean duration of successive cell generations, by a rapidly increasing loss of cells from the generative population, or by some combination of these processes that would result in a rapid deceleration of the growth of the tumor according to the function described below (Equation 5). Evidence bearing on this point will be presented in the discussion.

An exact mathematical description of our model of tumor cell proliferation is given by a Gompertz equation of the following form:

$$W/W_0 = e^{\frac{A}{\alpha}(1-e^{-\alpha t})} \quad (1)$$

where $W$ = tumor size, in appropriate units, at any time $t$, $W_0$ = initial tumor size, and $A$ and $\alpha$ are constants.

If, in Equation 1, we express $e^{-\alpha t}$ as a power series in $\alpha t$, we see at once that for small values of $\alpha t$, the growth function (Equation 1) reduces to

$$W/W_0 = e^{At} \quad (2)$$

i.e., simple exponential growth. This fact has two practical corollaries: (1) In the case that $\alpha$ is finite, i.e., where a retarding effect is operating on the growth of the tumor, $\alpha t$ will be small initially, when $t$ is still vanishingly small, and thus tumor growth will begin as simple exponential growth and will deviate from this more and more as time goes on. (2) In the special case that there is no retarding effect throughout the growth of the tumor, $\alpha$ is equal to zero, and the growth of the tumor will be exponential throughout. Thus this mathematical model includes simple exponential growth as the special case in which $\alpha$ happens to be zero.

In Table I are given the values computed for the Gompertzian constants $A$, $\alpha$, and $W_0$, for a number of the tumors reported in the literature. In all cases an arbitrary small value was first assigned to $W_0$ to obtain an estimate for the values of $A$ and $\alpha$. It was then possible to compute a value for $W_0$ by assigning these estimates as starting values, and computing the three parameters simultaneously by successive approximations. The data were weighted by the reciprocal of the tumor size for each point. For all of these operations, the computer was programmed to find the values for $A$, $\alpha$, and $W_0$ that would give the Gompertzian equation with the best fit to the experimental data, on the basis of least squares.

The values for $A$ lie generally between 0·08 and 0·36, and for $\alpha$ between 0·01 and 0·02, but several exceptions stand out, notably the high values for $A$ and $\alpha$ found for the Krebs tumor, and the very low value for $\alpha$ found for one of the Walker tumors. The ratio $A/\alpha$, which determines the asymptote of the growth curve, is remarkably similar in spite of the differences in the individual values for $A$ and $\alpha$.

TABLE I.—*Gompertzian Analysis of Tumor Growth*

Values for constants $A$, $\alpha$, and $W_0$, and upper limits of growth

| Tumor | Reference | A | $\alpha$ | $W_0$ | Theoretical Upper limit | Approximate Size at death |
|---|---|---|---|---|---|---|
| *Mouse:* | | | | | | |
| Krebs | (1) | $5 \cdot 25 \pm 2 \cdot 00$ | $0 \cdot 411 \pm 0 \cdot 056$ | $2 \cdot 7 \times 10^3$ cells | $1310 \times 10^6$ cells | $800 \times 10^6$ cells |
| Ehrlich | (2) | $0 \cdot 078 \pm 0 \cdot 011$ | $0 \cdot 009 \pm 0 \cdot 0008$ | $426 \times 10^3$ cells | $2500 \times 10^6$ cells | $1593 \times 10^6$ cells |
| $MC_1M$, low dose | (2) | $0 \cdot 119 \pm 0 \cdot 004$ | $0 \cdot 0147 \pm 0 \cdot 0015$ | $139 \times 10^3$ cells | $427 \times 10^6$ cells | $467 \times 10^6$ cells |
| $6C_3HED$, high dose | (3) | $0 \cdot 0397 \pm 0 \cdot 003$ | $0 \cdot 012 \pm 0 \cdot 0015$ | $50 \times 10^6$ cells | $1340 \times 10^6$ cells | $890 \times 10^6$ cells |
| $6C_3HED$, low dose | (3) | $0 \cdot 0626 \pm 0 \cdot 0062$ | $0 \cdot 0116 \pm 0 \cdot 0021$ | $10 \times 10^6$ cells | $2190 \times 10^6$ cells | $776 \times 10^6$ cells |
| DBA lymphoma | (3) | $0 \cdot 276 \pm 0 \cdot 023$ | $0 \cdot 0238 \pm 0 \cdot 0021$ | $10 \times 10^3$ cells | $1070 \times 10^6$ cells | ($1000 \times 10^6$ cells)* |
| $El_4$, low dose | (3) | $0 \cdot 207 \pm 0 \cdot 096$ | $0 \cdot 019 \pm 0 \cdot 003$ | $24 \times 10^3$ cells | $1290 \times 10^6$ cells | $1260 \times 10^6$ cells |
| $El_4$, high dose | (3) | $0 \cdot 172 \pm 0 \cdot 097$ | $0 \cdot 023 \pm 0 \cdot 004$ | $695 \times 10^3$ cells | $1240 \times 10^6$ cells | $1290 \times 10^6$ cells |
| EO771 | (4) | $0 \cdot 666 \pm 0 \cdot 304$ | $0 \cdot 063 \pm 0 \cdot 022$ | 3 $mm^3$ | 109 $cm^3$ | 31 $cm^3$ |
| Osteosarcomas | (5) | $1 \cdot 02 \pm 0 \cdot 115$ | $0 \cdot 159 \pm 0 \cdot 026$ | $0 \cdot 01$ $cm^3$ | $6 \cdot 03$ $cm^3$ | $4 \cdot 3$ $cm^3$ |
| *Rat:* | | | | | | |
| Walker, W26b1 | (6) | $0 \cdot 220 \pm 0 \cdot 0227$ | $0 \cdot 0218 \pm 0 \cdot 0061$ | $0 \cdot 4$ g | 9600 g | 175 g |
| Walker, W12a7 | (7) | $0 \cdot 342 \pm 0 \cdot 040$ | $0 \cdot 0205 \pm 0 \cdot 0058$ | $4 \cdot 2$ $mm^3$ | 72,800 $cm^3$ | 212 $cm^3$ |
| Walker, W10a6 | (7) | $0 \cdot 362 \pm 0 \cdot 017$ | $0 \cdot 039 \pm 0 \cdot 0037$ | 418 $mm^3$ | 1780 $cm^3$ | 490 $cm^3$ |
| Walker, W10b4 | (7) | $0 \cdot 132 \pm 0 \cdot 012$ | $0 \cdot 003 \pm 0 \cdot 0026$ | $16 \cdot 7$ $mm^3$ | — | 196 $cm^3$ |
| R39 Sarcoma, R3a7 | (8) | $1 \cdot 28 \pm 0 \cdot 250$ | $0 \cdot 124 \pm 0 \cdot 011$ | $8 \cdot 36$ $mm^3$ | 241 $cm^3$ | 188 $cm^3$ |
| R39 Sarcoma, R4c4 | (8) | $0 \cdot 540 \pm 0 \cdot 120$ | $0 \cdot 078 \pm 0 \cdot 012$ | 475 $mm^3$ | 496 $cm^3$ | 276 $cm^3$ |
| R39 Sarcoma, a7R3 | (8) | $0 \cdot 737 \pm 0 \cdot 162$ | $0 \cdot 063 \pm 0 \cdot 0068$ | $2 \cdot 1$ $mm^3$ | 270 $cm^3$ | 202 $cm^3$ |
| Flexner-Jobling | (9) | $0 \cdot 394 \pm 0 \cdot 066$ | $0 \cdot 049 \pm 0 \cdot 0063$ | $0 \cdot 015$ g | $48 \cdot 4$ g | $18 \cdot 3$ g |
| *Rabbit:* | | | | | | |
| Brown-Pearce | (8) | $1 \cdot 262 \pm 0 \cdot 270$ | $0 \cdot 169 \pm 0 \cdot 0168$ | 18 $mm^3$ | $31 \cdot 4$ $cm^3$ | $29 \cdot 8$ $cm^3$ |

Literature references : (1) Patt and Blackford, 1954 ; (2) Klein and Révész, 1953 ; (3) Révész and Klein, 1954 ; (4) Ting, 1952 ; (5) Finkel, Bergstrand, and Biskis, 1961 ; (6) Schrek, 1936*a* ; (7) Schrek, 1935 ; (8) Schrek, 1936*b* ; (9) Sugiura and Benedict, 1920.

* This is the value for the last two experimental points on the curve ; terminal scatter of the data included two previous points that were higher, 1290 and 1620 × $10^6$ cells.

Although the absolute values computed for $W_0$ in many cases seem large, they are extremely small on the scale of the growth process as a whole; except for the $6C_3HED$ lymphoma given at high dose, the values computed for $W_0$ are not greater than 0·1 per cent of the cell numbers finally approached in the growth of these tumors. That is, these curves cover in nearly all cases more than a 1000-fold increase in size of the tumor. $W_0$ is an extrapolated value, lying far removed from the data points, and hence has a very large standard error. For this reason, the values for $W_0$ can be accepted as only suggestive ; however, they are found to be reasonable values when the data on the authors' original plots are extrapolated back to the ordinate, in cases where this is possible. $W_0$ may be interpreted as indicating, however roughly, the initial effective growth mass of the tumor. The values are always much smaller than the initial dose of the tumor, where this figure is known, suggesting that many of the inoculated tumor cells die, as would be expected, leaving a relatively small number to establish the growing tumor.

The instability of the computed $W_0$ does not affect the rest of our analysis, however, because the essential parameters, $A$ and $\alpha$, and their standard errors, change little when $W_0$ is deliberately varied over a relatively wide range.

A Gompertz function of the form used here, as $t$ gets very large, approaches an asymptote whose value is given by the expression

$$W / W_0 = e^{A/\alpha} \quad (3)$$

The theoretical upper limit of size of these tumors, based on the computed asymptote of the Gompertz function, is given in column 6 of Table I, and in column

7 is given the size actually reached by the tumor before the death of the host. Considering at first only the mouse ascites tumors (all the mouse tumors except the EO771 and the primary osteosarcomas), we see that the theoretical upper limit of tumor size varies only about 5-fold, even though the values for $W_0$ vary about 1500-fold. To compare the limit of size of the solid tumors with that of the ascites tumors, it is necessary to express the asymptote in similar units, i.e., in terms of the total number of tumor cells. We can estimate this number on the basis of previous findings: Our own earlier studies establishing a common cell fractionation pattern for tumours (Laird, 1954 ; Laird and Barton, 1956) included estimates of the number of cells per gram in a number of tumors of several species. Two solid tumors of the mouse had a concentration of about 750 million cells per gram of tumor ; in many rat tumors the concentration of cells was about 400 to 500 million cells per gram. If these figures are incorporated into our present calculations, assuming a specific gravity for tumor tissue of 1, then the theoretical limiting size of the osteosarcomas is about 2400 to 4500 million cells. This figure is only slightly higher than those for the ascites tumors. On the other hand, the growth curve of the EO771 approaches a much higher upper limit than do those of the other mouse tumors studied ; on the basis of the above calculations, the upper limit, expressed in terms of total cell number, would be approximately 50,000 to 75,000 million cells, if the cells were of similar size. Considered together, however, the computed upper limits of the mouse tumors appear to fall within a relatively narrow range. Furthermore, for the mouse tumors the actual growth achieved before the death of the host is usually a high proportion of the theoretical limit of growth.

In contrast to the mouse tumors, the rat tumors show no evidence of constancy of the theoretical upper limit of size, and indeed the asymptotes calculated for the Walker tumor curves are so large as to be biologically meaningless. The size actually reached by the rat tumors before the death of the host is, however, much more nearly constant, the range lying between about 200 and 300 grams or c.c. for all the tumors except the Flexner-Jobling, which in the study included here reached only about 18 grams, and one of the Walker tumors, the W10a6, which grew to about 480 c.c.

The Brown-Pearce tumor of the rabbit, at least in the example chosen for computation, reached approximately the theoretical upper limit before the death of the host.

The Gompertz function is not usually considered in terms of doublings and doubling times, and the relation of the constants $A$ and $\alpha$ to the doubling process is not immediately evident. However, because our model of tumour growth is conceived in terms of doublings of over-all tumor size, and possibly also in terms of mean generation times of proliferating tumor cells, it is useful in this context to transform the information given by the Gompertz function into doublings and doubling times. It must be emphasized that these are interpretations of the theoretial functions alone, and are " true " for the tumors only to the extent that one accepts the Gompertz function as a valid representation of the realities of tumor growth.

The growth equation (Equation 1) can be rearranged as follows to give $t$ as a function of $W/W_0$:

$$t = \frac{1}{\alpha} \ln \left[\frac{A}{\alpha(A/\alpha - \ln W/W_0)}\right] \qquad (4)$$

The times required for the first doubling of tumor size, as estimated from the corresponding theoretical functions, are given in Table II, column 2. The tumors included in this study fall into several more or less homogeneous classes with respect to the initial doubling times. The shortest times, about 2·5 to 6 hours, were observed for the ascitic mouse tumors. The solid mouse tumors and the rat tumors, which were also solid, usually required about 1 to 5 days for the first doubling. The two exceptional ascitic tumors, the $6C_3HED$ lymphomas, which had been given at doses that were high relative to the theoretical and physiological upper limits of size for mouse ascites tumors, grew very slowly from the start; about 12 hours was required at the low dose and 20 hours at the high dose to double the initial effective tumor mass.

TABLE II.—*Analysis of Theoretical Gompertz Functions in Terms of Doublings*

| Gompertz curve Corresponding to: | Duration, initial doubling, | Ratio, second to first doubling time | Approx. No. doublings to upper limit |
|---|---|---|---|
| Krebs | 3·26 hours | 1·06 | 18 |
| Ehrlich | 9·26 hours | 1·10 | 12 |
| $MC_1M$, low dose | 6·09 hours | 1·11 | 11 |
| $6C_3HED$, high dose | 19·6 hours | 1·44 | 4·5 |
| $6C_3HED$, low dose | 11·9 hours | 1·19 | 7·5 |
| DBA lymphoma | 2·59 hours | 1·07 | 16 |
| $El_4$, low dose | 3·46 hours | 1·08 | 15 |
| $El_4$, high dose | 4·23 hours | 1·12 | 10 |
| EO771 | 1·08 days | 1·09 | 15 |
| Osteosarcomas | 5·03 days | 1·15 | 9 |
| Walker, W26b1 | 3·26 days | 1·08 | 14 |
| Walker, W12a7 | 2·07 days | 1·05 | 23 |
| Walker, W10a6 | 1·99 days | 1·09 | 13 |
| Walker, W10b4 | 5·29 days | 1·02 | 62 |
| R39, R3a7 | 0·56 days | 1·08 | 14 |
| R39, R4c4 | 1·35 days | 1·13 | 9·5 |
| R39, a7R3 | 0·97 days | 1·07 | 16 |
| Flexner-Jobling | 1·84 days | 1·11 | 11 |
| Brown-Pearce | 0·576 days | 1·12 | 10 |

A significant degree of retardation occurs in these Gompertz functions by the second doubling of $W/W_0$; the ratio of the duration of the second to the first doubling is given in column 3 of Table II. A prolongation of 2 to 10 per cent is evident by the second doubling for most of these functions, and is as great as 44 per cent in the case of the function corresponding to the $6C_3HED$ lymphoma, high dose.

This retardation increases as growth continues; the rate of increase in retardation is given by the following equation, in which the doubling time, $\Delta t$, is expressed as a function of time $t$:

$$\Delta t = -\frac{1}{\alpha} \ln \left[1 - \frac{\alpha \ln 2}{A} e^{\alpha t}\right] > 0 \qquad (5)$$

It is evident from this expression that successive doubling times will increase slowly at first while $t$ is small and will increase more and more rapidly as $t$ becomes large*. Correspondingly, the slowing of tumor growth described by a Gompertz

* If $A < \alpha \ln 2$, there is no doubling since the final value of $W/W_0$, $e^{A/\alpha}$, is less than 2. If $A > \alpha \ln 2$, the right hand side of Equation (5) has meaning only for

$$t < t^* = \frac{1}{\alpha} \ln \left[\frac{A}{\alpha \ln 2}\right].$$

At time $t = t^*$, $W/W_0$ equals $1/2\ e^{A/\alpha}$.

function is evident as a relatively small delay very early, which then increases in a continuously accelerated fashion.

The Gompertzian growth equation (Equation 1) indicates that the value of $W/W_0$ increases from the value 1 at time zero to the final value $e^{A/\alpha}$ at the asymptote, i.e., ln $W/W_0$ increases from 0 to $A/\alpha$. Since one doubling represents an increment of ln2 in ln $W/W_0$, the total number of doublings ($n$) from time zero to reaching the asymptote is given by the expression

$$n = \frac{A}{\alpha \,.\, \ln 2} \tag{6}$$

It is clear that $n$ is directly related to the ratio of $A$ to $\alpha$ ; i.e., the larger the ratio $A/\alpha$, the greater the number of doublings that will be possible before the Gompertz function reaches its asymptote. A Gompertz function corresponding to the $6C_3HED$ lymphoma, high dose, with $A/\alpha = 3{\cdot}3$, will pass through only about 4·5 doublings, while a Gompertz function corresponding to the Walker tumor, W10b4, with $A/\alpha = 44$, will permit 62 doublings to take place before the upper limit is reached.

An example of a Gompertz plot of tumor growth, that of the Krebs ascites carcinoma, is shown in Fig. 4. This figure also includes an exponential curve, illustrating the course tumor growth would have followed if no retardation had occurred. For comparison, a curve of bacterial growth is also shown ; the data for this curve were obtained by automatic counting of a culture of *E. coli* during its growth (data kindly given us by H. Kubitschek, of the Argonne National Laboratory). Such bacterial growth also deviates from exponential growth, as environmental conditions become limiting. However, when the fit of bacterial growth to a Gompertz function was tested, the Gompertzian model of growth was found to be statistically incompatible with the data. This is to be expected, because, as stated above, the retardation described by a Gompertz function increases very slowly at first, and then faster and faster, according to Equation 5 ; the retardation observed when bacterial cultures approach a limit is, compared to a Gompertzian retardation, very abrupt, changing quickly from essentially zero in the logarithmic phase of growth to an infinitely large value as growth stops.

### DISCUSSION

The expectation that tumor growth under ideal conditions would prove to be exponential until it terminates with the exhaustion of the host has not been borne out in many careful studies of the growth of a wide variety of tumors. The deviation from a semi-log line is not just a terminal finding ; in most cases tumor growth is smoothly curvilinear on a semi-log plot throughout observed growth. This finding implies directly that the specific growth rate of tumors is usually not constant even for a short time, but decreases steadily.

In the present study we have shown that tumor growth is well described by a Gompertz function, according to which the times required to double the tumor mass increase according to an exponential function. Other functions might be used that would fit the data equally well, but all would necessarily be functions in which the doubling times become longer at a continuously increasing rate, because the tumor data themselves show this property. The Gompertz function used here was chosen because it gives an exact mathematical description of a useful model of cell proliferation, one in which cells are regarded as multiplying exponentially, but their

FIG. 4.—An arithmetic plot of (1) the theoretical Gompertz curve giving the best fit by the method of least squares to the experimental data, Krebs ascites carcinoma. The circles are the original experimental points. Data of Patt and Blackford (1954). (2) Growth curve of *Escherichia coli*, B/r, grown in broth culture.* (3) An exponential curve fitted to the early growth data, showing the course growth would have taken if no retardation had occurred in either the bacterial culture or the tumor. The small scale of the graph obscures the fact that the early experimental points for the tumor also deviate from the exponential curve, as would be necessary to allow us to compute an upper limit of growth on the basis of a Gompertz function.

* Data kindly given us by H. Kubitschek, of the Argonne National Laboratory.

net accumulation is subjected to some retarding factor(s) whose effect increases during growth according to an exponential function. This retarding effect might be due to an increase in mean generation times without a change in the proportion of reproducing cells, or it might be due to a loss in reproductive cells without change in the mean generation time of the cells, or it is possible that these two factors might be conbined in such a way as to result in the observed slowing of tumor growth according to an exponential function.

For a loss of cells to account entirely for the observed retardation, such loss would have to increase in successive generation times, according to Equation 5. A loss of cells that remained constant per generation time would merely depress the growth rate, leaving the growth process semi-logarithmic, since the remaining viable cells would still increase in geometric progression ; the growth curve would therefore still be a straight line on a semi-log plot, not the curved line that is actually observed. In a search for dying cells as a possible explanation for the deviation from semi-log growth observed in their own work, both Klein and Révész (1953), studying the Ehrlich ascites tumor, and Patt and Blackford (1954), studying the Krebs ascites carcinoma, noted that the proportion of non-viable cells in these tumors is very low, and does not change significantly with time. Unless we assume that an exponentially increasing loss of cells was completely obscured by the immediate removal of dying cells from the population, the data do not suggest that cell loss alone was the cause of the decreasing rate of exponential growth. Furthermore, in essential agreement with these results, Baserga, Kisieleski, and Halvorsen (1960) found nearly 100 per cent of the cells of an Ehrlich tumor labeled by titrated thymidine when the tumor was exposed to the label at intervals over a period equal to only a single generation time ; they also found that the per cent of cells labeled 4 hours after a single injection of tritiated thymidine did not change significantly between the second and eleventh day of tumor growth. In contrast to these findings with transplanted tumors, the results of Mendelsohn's (1962) labeling experiments with an autochthonous mammary tumor of the mouse suggested the presence of a significant fraction of non-proliferating cells in the tumor. It should be noted in passing that if slowing of tumor growth were entirely due to an increase in generation times with no change in the proportion of viable cells, a reduction in the per cent of labeled cells would ultimately be detected as tumor growth progressed, if the duration of DNA synthesis remained constant ; if, on the other hand, the duration of DNA synthesis increased in proportion to the increase in generation time, no change in the per cent of labeled cells would be observed. In any case, Mendelsohn's data do not allow us to determine whether any change in the proportion of non-proliferating cells occurred with time, and as we noted above, a constant proportion of non-proliferating cells would merely depress the rate of a semi-log growth process, but would not in itself produce the deceleration actually observed in tumor growth.

Although necrosis is evident in the majority of solid tumors, especially if growth is prolonged, dead cells and their proteins tend to remain *in situ*, as shown by Reid and White (1959) in a radioautographic study of several rat and mouse tumors, and hence the earlier contribution to tumor size remains ; the only loss is in the proportion of reproductive cells contributing to later tumor growth. However, this loss, to account by itself for the observed deviation from semi-log growth, must be more than a constant loss per generation, must in fact be an exponentially increasing loss, as discussed above. Hence, considering the data available at the present time, it seems likely that the observed deceleration of tumor growth is due at least in part to an actual increase in the mean generation time during tumor growth.

A Gompertz function of the form used here is a function with a horizontal asymptote. The upper limit of growth as computed for these tumors is a theoretical projection based on the measured growth of the tumor. For all the tumors included in the present survey, enough data were presented in the original studies to allow us to fit such a function to the data, and to project the theoretical upper limit of growth. For most of the tumors the growth actually observed before the death of the host was a large fraction of the projected growth, falling short of the asymptote by only one or at most two doublings of tumor size. If we were to confine our attention to these cases alone, we would probably conclude that the slowing of tumor growth simply reflects a terminal failure of the host to give nutritional support to the tumor. However, for several of the tumors, the observed growth fell far short of the projected upper limit, although the tumors all reached about the same size before the death of the host. Stated conversely, the projected limit of size for the latter tumors was very much greater than any tumor could possibly reach, and hence little retardation was observed even during the terminal illness and death of the host. Nevertheless, we would not be able to compute an upper limit for the latter group of tumors if some retardation were not indicated by the data even this early in the growth process. These facts together suggest that the marked slowing of growth that is observed terminally for many tumors is not simply due to failure of the host to support growth, but is a product of host-tumor interaction that is evident from earliest tumor growth.

A further argument that the retardation of tumor growth is not due to simple passive failure of the host to provide nutritional support is based on the difference between the mathematical nature of the tumor retardation and the slowing of growth seen when bacterial cells grow in a closed system, with a fixed nutritional limitation. In the later case, so long as an excess of nutrient is present, the cells proliferate exponentially ; relatively suddenly the concentration of nutrient is reduced below a threshold value by the metabolic activity of the bacterial cells, whereupon growth slows rapidly and soon ceases. In such cases, the limit of growth is usually a linear function of the concentration of the limiting nutrient in the surrounding medium (see Monod, 1949). It seems probable, although we have not tested the hypothesis, that to force bacterial growth to follow a Gompertzian model, it would be necessary to grow such bacteria in a chemostat-like environment, with a progressively increasing reduction of the limiting nutrient, in addition to the exhaustion of nutrient by the bacteria themselves. In other words, the retardation of growth described by a Gompertzian model appears to be an actively increasing depression of the specific growth rate, rather than a passive, pre-set limitation imposed by exhaustion of the available growth-supporting factors in the environment.

Both for this reason, and because even the terminal illness and death of the host does not produce extensive retardation in the growth of a tumor if it is still well below the theoretical upper limit of size, it seems probable that growth of tumors in an animal host meets some more active resistance than just a failure of the blood supply, etc. The biological nature of such resistance is not demonstrated in the present mathematical analysis of tumor growth ; it might be an immune response (but not just immunity to transplanted tumors, because retardation of the characteristic type is seen as well in primary tumors as in transplanted tumors), or it might be some as yet poorly understood " feedback " control exerted by the whole organism on the growth of its parts, to which the tumors are still responsive to a greater or lesser extent.

Yet a physiologic limit to tumor growth does seem relatively constant ; the host can tolerate so much growing tumor, and then dies. If the physiologic limit and the theoretical limit of tumor growth are in some cases independent, and if some of the retarding factors are active, as posited above, it is then conceivable that the growth-retarding factors might be artificially stimulated, producing a slowing of tumor growth toward a theoretical limit that is less than the physiologic limit imposed by the tolerance of the host. Carcinoma *in situ* might be an example of a normal occurrence of such a phenomenon ; indeed, Dunn (1961) reports on the basis of two epidemiological studies that the incidence of carcinoma *in situ* of the human cervix uteri is in excess of that required to produce the known incidence of invasive cancer, suggesting that some carcinomas *in situ*, perhaps as many as one-third to one-half the total, fail to progress to invasive carcinoma.

Search for natural growth-regulating mechanisms is of practical importance, because the clinical disease of cancer in both animals and humans, i.e., the systemic illness and death of the host, is limited to the time of the last two or three doublings of tumor size. Thus for most tumors a relatively small stimulation of growth-retarding factors might prevent a tumor from reaching the physiologic limit of host survival, with the consequence that the systemic illness might be delayed, or even in some cases eliminated, if the delay should exceed the normal life-expectancy of the host.

SUMMARY

The growth of nearly all tumors reported in the literature is characterized by a continuous deceleration from the earliest periods of observation. Growth of this nature is well described by a Gompertz function of the form :

$$W/W_0 = e^{\frac{A}{\alpha}(1-e^{-\alpha t})}$$

in which $W_0$ = initial tumor size, $W$ = tumor size at time $t$, and $A$ and $\alpha$ are constants.

Such a function has been fitted to the growth data of a number of tumors in the mouse, rat and rabbit, and has been shown to follow growth through a 1000-fold increase in tumor size.

A Gompertz function of this type has a horizontal asymptote ; this fact implies that growth of tumors progresses toward an upper limit of size. The upper limits computed for the mouse tumors fell within a relatively narrow range, and the size actually achieved by the tumor before the death of the host was usually a high proportion of the theoretical limit of growth In contrast, the theoretical limits for the rat tumors were usually so high as to be biologically meaningless ; however, a relatively constant physiological limit was placed on tumor size by the death of the host. In these cases, no terminal increase in retardation was observed, even in a dying host.

For several reasons, the observed retardation of tumor growth appears to be due to an actively increasing depression of the growth rate. Stimulation of natural growth-retarding factors might be of practical importance in reducing the incidence of clinical cancer, i.e., the systemic illness and death of the host, if tumor growth could be retarded enough to bring it to an upper limit that is within the physiological tolerance of the host.

The author is grateful to Sylvanus A. Tyler and Merlin H. Dipert of this department for their generous assistance in the mathematical analysis.

This work was supported by the United States Atomic Energy Commission.

### REFERENCES

Baserga, R., Kisieleski, W. E. and Halvorsen, K.—(1960) *Cancer Res.*, **20**, 910.

Dunn, J. E., Jr.—(1961) *Proceedings of the IVth Berkeley Symposium on Mathematical Statistics and Probability*, **4**, 211.

Finkel, M. P., Bergstrand, P. J. and Biskis, B. O.—(1961) *Radiology*, **77**, 269.

Klein, G. and Révész, L.—(1953) *J. nat. Cancer Inst.*, **14**, 229.

Laird, A. K.—(1954) *Exp. Cell Res.*, **6**, 30.—(1962) *Proc. Amer. Ass. Cancer Res.*, **3**, 336.

*Idem* and Barton, A. D.—(1956) *Science*, **124**, 32.

MAYNEORD, W. V.—(1932) *Amer. J. Cancer*, **16**, 841.
MENDELSOHN, M.—(1962) *Science*, **135**, 213.
MONOD, J.—(1949) *Annu. Rev. Microbiol.*, **3**, 371.
PATT, H. M. AND BLACKFORD, M. E.—(1954) *Cancer Res.*, **14**, 391.
REID, J. C. AND WHITE, J.—(1959) *J. nat. Cancer Inst.*, **22**, 845.
RÉVÉSZ, L. AND KLEIN, G.—(1954) *Ibid.*, **15**, 253.
SCHREK, R.—(1935) *Amer. J. Cancer*, **24**, 807.—(1936*a*) *Amer. J. Path.*, **12**, 525.—(1936*b*) *Amer. J. Cancer*, **28**, 345.
SCHWARTZ, M.—(1961) *Cancer*, **14**, 1272.
SUGIURA, K. AND BENEDICT, S. R.—(1920) *J. Cancer Res.*, **5**, 373.
TING, T. P.—(1952) *Science*, **116**, 149.